# High volume retrograde portography for better discrimination of the portal vein during TIPS procedure

**DOI:** 10.1177/20584601221128405

**Published:** 2022-09-20

**Authors:** J Altenbernd, S Zimmer, L Andrae, B Labonte, J Gruber, H Beier, M Abdulgader, M Buechter, M Forsting, J Theysohn

**Affiliations:** 1 Institute of Diagnostic and Interventional Radiology and Neuroradiology, 39081 University Hospital Essen, Germany; 2 Institute of Radiology and Neuroradiology, 39081 Gemeinschaftskrankenhaus Herdecke, Germany; 3 Institute of Radiology and Neuroradiology, St Marien-Hospital Hamm, 39081 Gemeinschaftskrankenhaus Herdecke, Germany; 4 Internal Medicine and Gastroenterology, 39081 Gemeinschaftskrankenhaus Herdecke, Germany; 5 Internal Medicine and Gastroenterology, 14883 Allgemeines Krankenhaus Hagen, Germany; 6 Internal Medicine and Gastroenterology, St Elisabeth Hospital Iserlohn, Germany

**Keywords:** TIPS, portal vein puncture, retrograde portography

## Abstract

**Background:** Imaging of the portal vein prior to puncture for TIPS is essential.

**Purpose:** With this study, we examined a modified retrograde portography with regard to the reliable representation of the portal vein.

**Material and Methods:** Prospective evaluation of 65 TIPS interventions with regard to the delimitation of the portal vein and the exact parameters of retrograde portography such as catheter diameter and contrast medium volume per injection.

**Results:** Retrograde portographies with a large-lumen catheter (10 F) and a large contrast medium volume (40 mL) were performed in 35/63 patients with significantly better delineation of the portal vein than when using 5 F catheters with 10 mL contrast medium.

**Conclusion:** The so-called high volume retrograde portography leads to better delimitation of the portal vein during TIPS application.

## Introduction

The creation of a transjugular portosystemic shunt (TIPS) is an established method for the treatment of portal hypertension.^1,2^ One of the challenges during TIPS intervention is the puncture site of the portal vein, usually the right main trunk of the portal vein.^3,4^ Visualization of the portal vein system before and during the puncture is therefore important and minimizes risk of intraprocedural complications. In addition to pre-interventional computed tomography, peri-interventional ultrasound, indirect portography after arterial contrasting, or retrograde portography help to visualize the portal vein. Retrograde portography can again be performed with a contrast medium containing iodine or carbon dioxide.^5–7^ Percutaneous wire placement or Cone-Beam CT as guiding methods for portal vein delineation are also possible.^8,9^ With this prospective study, we examined to what extent the quality of retrograde portography with contrast media can be influenced by optimizing individual parameters.

## Material and methods

Between January 2019 and September 2021, we performed 115 retrograde portographies in 65 patients; all of them subsequently underwent a TIPS procedure for decompensated portal hypertension. In our institute, we deliberately carry out a retrograde portography without a wedge as standard. Our experience shows that there is a lower risk of laceration.

Three different retrograde portography protocols were compared prospectively, protocol 1 with injection of 10 mL of diluted (1:1 with saline) contrast medium with a 10 mL syringe via a 5 French multipurpose catheter, protocol 2 with injection of 20 mL of diluted (1:1 with saline) contrast medium with a 20 mL syringe via a 10 French guiding catheter, and protocol 3 with injection of 40 mL of diluted (1:1 with saline) contrast medium with a 50 mL syringe via a 10 French guiding catheter, respectively. All injections were performed manual. All procedures were performed within an angiography suite (Canon).

Two experienced interventional radiologists assessed the image quality with regard to the recognizability of the portal vein bifurcation and the left and right portal vein trunk (1-not recognizable; 2-incompletely recognizable; 3-completely recognizable).

**Figure 1.**
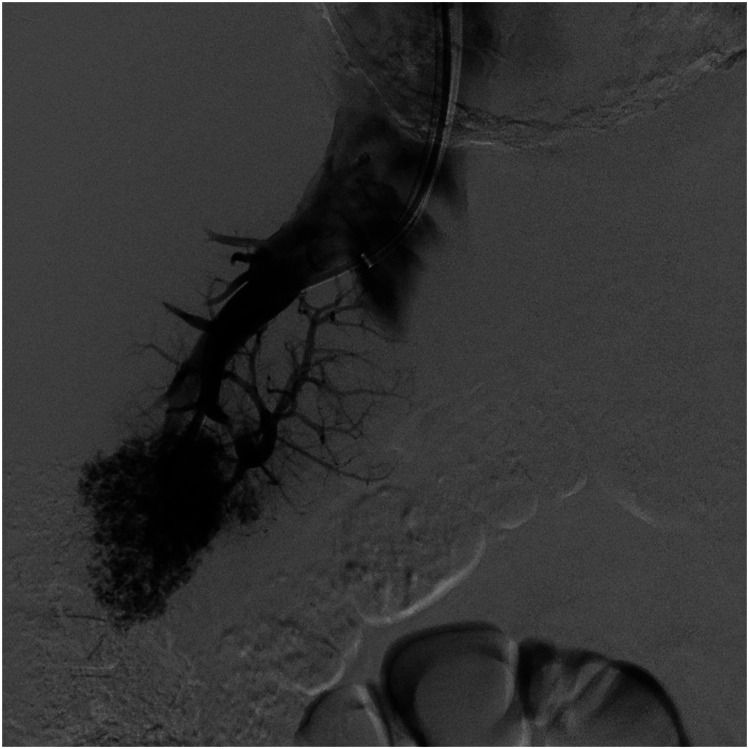
Retrograde portography performed with 10 mL iodinated contrast medium over a 5 French catheter.

All procedures performed in study were in accordance with the ethical standards of the institutional and with the 1964 Helsinki declaration and its later amendments or comparable ethical standards. Study informed consent was signed from each patient. Ethics vote has been obtained Figure 1.

## Results

**Figure 2.**
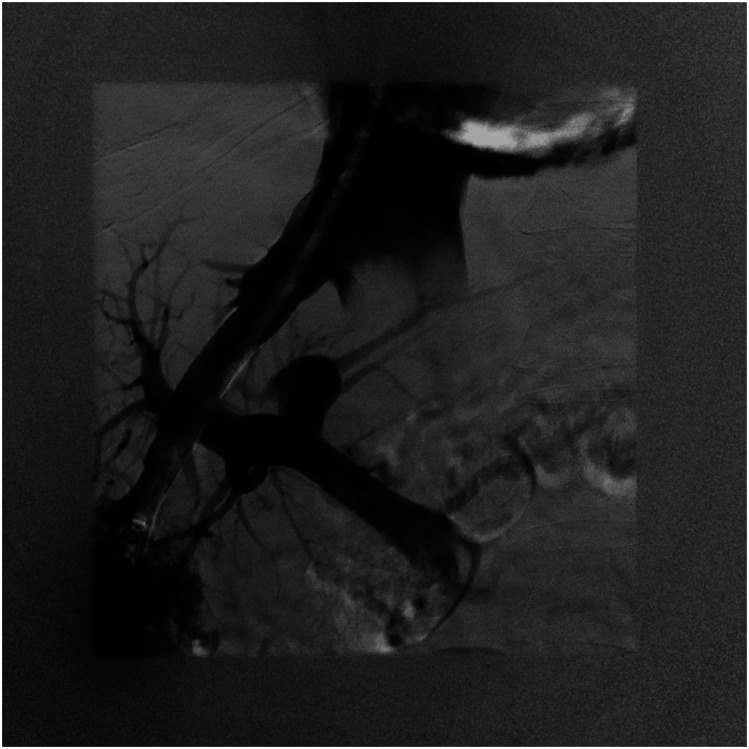
Retrograde portography performed with 40 mL iodinated contrast medium over a 10 French guiding catheter.

A total of 115 retrograde portographies were performed in 65 patients as part of the TIPS procedure. In 20 patients, retrograde portographies were carried out once, in 40 patients twice, and in 5 patients 3 times, respectively. Retrograde portographies were divided into the abovementioned three different injection protocols as follows: 36/115 protocol 1, 34/115 protocol 2 and 45/115 protocol 3. The average degree of recognizability of the portal bifurcation and the two main trunks was 2.3 +/− 0.4 for all 115 retrograde portgraphies with a minimum of 1 and a maximum of 3. For protocol 1, the result was a visibility of 1.9 +/− 0.5, for protocol 2, 2.1 +/− 0.4, and for protocol 3, 2.7 +/− 0.3, respectively. The difference between protocols 1 and 2 was not statistically significant (*p* = 0.1), whereas protocol 3 was associated with significantly better recognizability compared to protocol 1 (*p* <.05) and protocol 2 (*p* < .05) Figure 2.

## Discussion

The treatment of portal hypertension by transjugular creation of a portosystemic shunt has become increasingly important in recent years.^2,10,11^ Good visibility of the portal vein before puncture is essential for a low peri- and post-interventional risk in order to minimize the number of puncture attempts and avoid iatrogenic injuries to liver capsule, liver arteries, or biliary tract.

With the present study, we were able to prove that the image quality of retrograde portography with iodine-containing contrast media, in addition to anatomical and organ-specific influencing factors, depends on the inside diameter of the catheter used and the amount of contrast medium injected.

In our institute, we deliberately carry out a retrograde portography without a wedge as standard. Our experience shows that there is a lower risk of laceration.

In recent years, CO2 retrograde portography has become established in many centers since previous studies have shown positive results. However, in these cases, retrograde portographies were predominantly performed after injecting 40 mL of CO2, compared to classic retrograde portographies after injecting 10 mL of contrast medium containing iodine.^3–7,12^

So far, we are not aware of any study that examined retrograde portography protocols for contrast media containing iodine with regard to different catheter diameters and injection volumes.

Use of retrograded hepatic CO2 portography was introduced into the TIPS procedure by Rees and colleagues.^13^

The much better results in revealing the portal anatomy using CO2 instead of iodinated contrast medium are explained by the low viscosity of CO2 (more than 400 times lower than that of liquid contrast medium). The gas can better traverse the hepatic sinusoids in a volume large enough to cause temporary reversal of the portal vein blood flow. Gravity plays an important role in the distribution of injected gas in the portal vein.^5,6,13,14^

There are possible complications unique to CO2. Iodinated contrast medium mixes with blood, whereas CO2 displaces blood. Thus, when injected in large volumes, CO2 can displace the blood from the right heart and the main trunk of pulmonary artery, causing so-called vapor lock and resulting in transient hypotension and cardiac arrest.^7,15^

In our cohort, the use of a large-lumen injection catheter in combination with a larger, diluted amount of contrast medium led to better delimitation of the portal vein during TIPS procedure.

Interestingly, we did not detect any iatrogenic liver lacerations as a complication of the positive pressure injection in our collective, which is probably due to the manual administration of diluted contrast medium without an injector. In addition, the injection catheters were not advanced to the most distal point of the right hepatic vein.^16^

In conclusion, high volume injection for retrograde portography during TIPS intervention seems to be a good alternative if the requirements for the CO2 equipment are not met or the experience for ultrasound guided puncture is not available.
